# Clinical practice guidelines on hemochromatosis: Asian Pacific Association for the Study of the Liver

**DOI:** 10.1007/s12072-023-10510-3

**Published:** 2023-04-17

**Authors:** Darrell H. G. Crawford, Grant A. Ramm, Kim R. Bridle, Amanda J. Nicoll, Martin B. Delatycki, John K. Olynyk

**Affiliations:** 1 Faculty of Medicine, The University of Queensland, Brisbane, Australia; 2 Gallipoli Medical Research Foundation, Brisbane, Australia; 3 Hepatic Fibrosis Group, QIMR Berghofer Medical Research Institute, Brisbane, QLD Australia; 4 Department of Gastroenterology, Eastern Health, Box Hill, VIC Australia; 5 Monash University, Melbourne, VIC Australia; 6 Bruce Lefroy Centre, Murdoch Children’s Research Institute, Melbourne, VIC Australia; 7 The University of Melbourne, Melbourne, VIC Australia; 8 Victorian Clinical Genetics Services, Parkville, VIC Australia; 9 Department of Gastroenterology, Fiona Stanley Hospital, Murdoch, WA Australia; 10 School of Medical and Health Sciences, Edith Cowan University, Joondalup, WA Australia

## Introduction and summary

Hereditary hemochromatosis is the result of pathogenic variants in multiple genes that can result in increased body iron stores with excess iron deposited in various organs, including the liver, pancreas, and heart. The two most important advances in the field over the past 30 years have been the identification of the HFE gene (and the associated p.Cys282Tyr substitution), and the discovery of the hormone hepcidin, which is inappropriately low in this condition and is the pathophysiological basis of the increased iron absorption. The identification of mutations in the HFE gene and subsequent studies have reshaped diagnostic algorithms resulting in a marked reduction in the need for liver biopsy. The discovery of hepcidin has resulted in many studies that have dramatically improved our understanding of iron metabolism with clear potential therapeutic implications. The variable clinical expression of hemochromatosis has puzzled clinicians and scientists, and our understanding of the factors that influence the phenotype has increased over recent years. Nevertheless, increased clinician and patient awareness, early diagnosis, and therapeutic phlebotomy to restore normal life expectancy are still the cornerstones of management. The classic triad of cirrhosis, diabetes, and skin pigmentation is now uncommon, and many patients are diagnosed with minimal or no symptoms.

These guidelines have been developed to assist clinicians in the management of patients with hemochromatosis. They have been developed with the recent passing of Professor Lawrie Powell fresh in our minds. Professor Powell was one of the world’s leading authorities in the field of iron metabolism and hemochromatosis, and a co-founder of the Asian Pacific Association for the Study of the Liver. The authors dedicate these guidelines to the memory of Professor Powell in recognition of his remarkable contribution to knowledge in the field.

## Overview of iron metabolism

The total body iron pool of a normal adult is approximately 3–4 g, or 50 mg/kg. Most of this iron is located in erythrocytes as hemoglobin (60–70%) with the balance located in skeletal muscle, liver, spleen, and bone marrow as storage iron in ferritin and hemosiderin, iron-containing enzymes, and bound to transferrin. The average Western diet contains approximately 6 mg of iron per 1000 cal. Of this, only about 1–2 mg per day is absorbed by the duodenal mucosa.

**Fig. 1:**
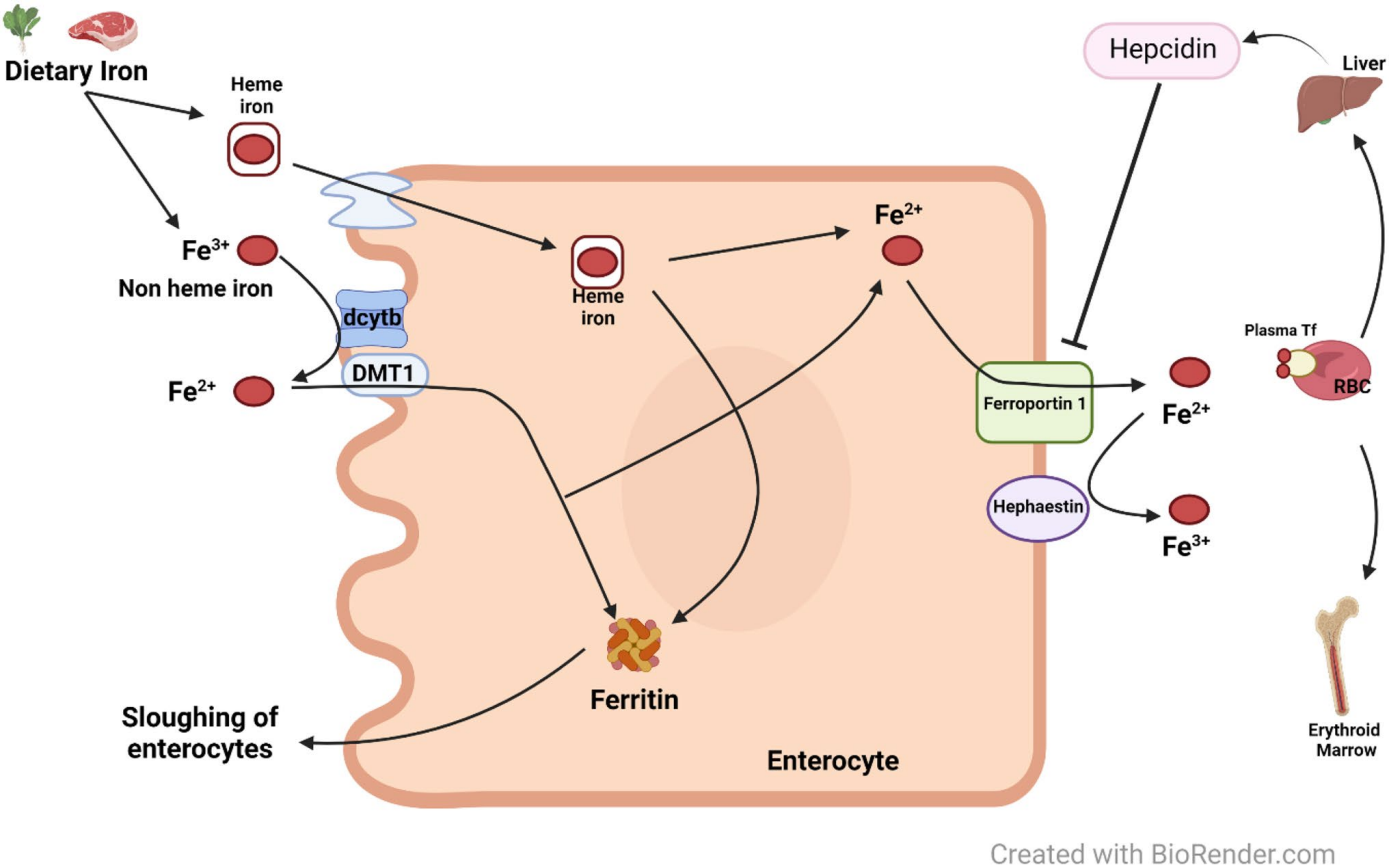
Intestinal Iron Absorption. Iron absorption occurs in the enterocytes of the small intestine before internalization of heme or non-heme iron. Ferrous iron is taken up by DMT1 following reduction by DCYTB. Excess iron is stored in ferritin or lost via enterocyte sloughing. Ferrous iron is exported via ferroportin, oxidized by hephaestin and transported by transferrin to various tissues for use or storage. Hepcidin senses body iron stores and acts as a negative regulator. Hepcidin binds to the iron export protein, ferroportin, on target cells and is responsible for the internalization and degradation of ferroportin. *DMT1* divalent metal transporter 1, *dcytb* duodenal cytochrome B, *RBC* red blood cell, *Fe*^*3+*^ ferric iron, *Fe*^*2+*^ ferrous iron, *Tf* transferrin

Iron absorption occurs in the enterocytes of the small intestine (predominately the duodenum and first section of the jejunum). Heme iron absorption is thought to occur via endocytosis, but little is known of this process [1]. Divalent metal transporter 1 (DMT1) is responsible for the uptake of ferrous iron (Fe^2+^) following reduction of ferric iron (Fe^3+^) by duodenal cytochrome B (DCYTB). Excess iron is then stored in ferritin or is lost through enterocyte sloughing. Ferrous iron is exported through ferroportin (FPN) on the basolateral membrane of the enterocyte and oxidized by hephaestin (Fig. 1). Approximately 80–85% of absorbed iron is transported by transferrin to the reticuloendothelial system or to bone marrow for incorporation into hemoglobin, stored in the liver or muscle, or used for heme synthesis [2].

The liver is an important organ in iron metabolism through its storage of iron and its synthesis of transferrin and hepcidin. The iron-sensing molecule, hepcidin, is responsible for sensing body iron stores and acts as a negative regulator. Increased iron levels result in increased hepcidin synthesis, which decreases release of iron into the circulation. Hepcidin binds to the iron export protein, ferroportin, on target cells and is responsible for the internalization and degradation of ferroportin. Patients with hereditary hemochromatosis have inappropriately reduced hepcidin expression due to mutations in *HFE* [3]. In addition to iron, infection, inflammation, and hypoxia can alter hepcidin expression [4].

## Hereditary hemochromatosis—classification, genetics, and epidemiology

### Genetics of hereditary hemochromatosis

Pathogenic variants in multiple genes can result in systemic iron overload. Table 1 lists a classification of hereditary hemochromatosis modified from recommendations by the Nomenclature Committee of the International Society for the Study of Iron in Biology and Medicine [5]. Systemic iron overload can occur in several other hereditary conditions that will not be considered further here. These include β-globin disorders, such as beta-thalassemia, sideroblastic anemia, pyruvate kinase deficiency, and hereditary spherocytosis.

**Table 1:** Modified Classification of Hemochromatosis based on Recommendations of the International Society for the Study of Iron in Biology and Medicine (modified from [5])

Novel classification	Molecular pattern	Features
*HFE*-related	p.Cys282Tyr homozygosity compound heterozygosity p.Cys282Tyr/ His63 Asp compound heterozygosity of p.Cys282Tyr with other rare pathogenic variants 106-109or *HFE* deletion.110	Variable penetrance Always consider the presence of host or environmental cofactors for iron overload and co-toxic liver injury, e.g., alcohol, metabolic-associated fatty liver disease In subjects with, p.Cys282Tyr/His63Asp compound heterozygosity or p.His63Asp homozygosity and iron overload-related disease consider referral to a specialist center to determine the need for second-line genetic testing for rarer variants
Non-*HFE*-related	Rare pathogenic variants in “non-*HFE*” genes: * HJV*-related * HAMP*-related * TFR2*-related * SLC40A1* (GOF)-related	May be associated with severe iron loading in multiple organs in younger populations While mutations in any hepcidin-regulatory gene may be causative, the effects of novel mutations should be confirmed through functional and epidemiological studies Molecular subtypes characterization only at specialized centers, but the diagnosis of non-HFE-related hemochromatosis is sufficient to start treatment at non-specialized centers
Digenic	Double heterozygosity and/or double homozygosity/heterozygosity for mutations in 2 different genes involved in iron metabolism (*HFE* and/or non-*HFE*)	More commonly, p.Cys282Tyr mutation in HFE gene might coexist with mutation in other genes; rarely, both mutations involve non-HFE genes
Molecularly undefined	Molecular characterization (still) not available after sequencing of known genes (provisional diagnosis)	The case should be referred to specialized centers for further consideration

Forms of both *HFE*-related and non-*HFE*-related hemochromatosis result in iron overload by diminished hepcidin. Iron overload can also be the result of impairment of the export function of ferroportin.

The age of onset of iron accumulation and therefore of symptom onset differs among the different genetic causes of hemochromatosis. The most common form of hereditary hemochromatosis is that due to p.Cys282Tyr homozygosity in HFE and manifests in adulthood. Males are more likely than females to develop iron overload and symptomatic disease. Compound heterozygous HFE-related hemochromatosis (p.Cys282Tyr/p.His63Asp) is usually clinically inconsequential but may cause liver injury and cirrhosis when accompanied by cofactors, such as regular moderate to high alcohol consumption, metabolic fatty liver disease, or hepatitis C infection.

However, other rarer forms of hemochromatosis are recognized and have different phenotypes. The HFE p.Ser65Cys substitution is not associated with excess iron storage in tissues or end organ injury and is therefore not of clinical significance. Juvenile hemochromatosis, which most commonly results from biallelic pathogenic variants in *HJV* or *HAMP*, has an onset of iron accumulation in early childhood with symptoms becoming evident as early as the first decade of life. It is often associated with severe iron overload with multiple organs involved. Males and females are affected equally by juvenile hemochromatosis. Hepcidin deficiency occurs due to pathogenic variants in the transferrin receptor 2 (TFR2) gene. Ferroportin disease occurs due to either reduced export function of ferroportin or to resistance of ferroportin to hepcidin.

### Epidemiology

Multiple studies have examined the frequency of hemochromatosis and the underlying genetic cause in different populations. These are summarized in Table 2. By far the most common cause of hemochromatosis is homozygosity for the p.Cys282Tyr substitution in the HFE protein. Compound heterozygosity for p.Cys282Tyr/p.His63Asp in HFE is more common than p.Cys282Tyr homozygosity but has markedly lower biochemical and clinical penetrance. Among individuals diagnosed with hemochromatosis, HFE p.Cys282Tyr homozygosity accounts for most in Australia [6] and 96% in Brittany, France [7], while only accounting for 62% in Italy [8] and 39% in Greece [9].

**Table 2:** Selected population studies of the frequency of HFE p.Cys282Tyr homozygosity

Country	Study population	Cohort size	Frequency of HFE p.Cys282Tyr
Australia [23]	Workplace	11,307	1 in 221
Australia [24]	Cohort enrolled through the electoral roll (enriched for Northern European ancestry)	29,676	1 in 146
USA [11]	Primary care and blood drawing laboratories	99,711	1 in 333
United Kingdom [25]	Postal invitation to individuals registered with the National Health Service	451,243	1 in 156
Norway [26]	Hospitalized individuals (Caucasian only)	1900	1 in 136
Spain [27]	Blood donors	5370	1 in 671
France [28]	Attendees at health appraisal centers	9396	1 in 174

The highest prevalence of HFE p.Cys282Tyr homozygosity is among Northern Europeans, in particular, Ireland and Scandinavia, with a lesser prevalence in Southern Europe [10]. HFE p.Cys282Tyr homozygosity is rare in those of African and Asian ancestry with estimates of prevalence being 1 in 6781 and 1 in 25,000, respectively [11, 12].

Hemochromatosis is a rare disorder in non-Caucasian populations. Isolated cases of C282Y homozygous hemochromatosis have occasionally been reported but the C282Y mutation has a very low frequency in the Asia Pacific region [13]. The H63D mutation is more common with a 2% allele frequency in Asian population compared with 15% in Europeans [13]. Other rare mutations in HFE including the E277K, Y231del and a homozygous splice mutation, IVS5 + 1 G > A have been reported [14–16]. Mutations in HJV and HAMP causing juvenile iron overload have also been reported throughout the Asia Pacific region but remain extremely uncommon. Compound heterozygous mutations of HJV or combined heterozygous mutations of the BMP/SMAD pathway genes leading to reduced hepcidin expression have been described in China [17]. Although rare, mutations in TFR-2 may be the leading cause of hemochromatosis in the Asia Pacific region where the I238M variant of TFR2 (previously reported as a polymorphism) has an allele frequency of 7% [18–20]. Ferroportin disease has a worldwide distribution with descriptions of associated iron overload in patients from the Solomon Islands, Sri Lanka, Vietnam, and India. Mutations in ferroportin that disrupt the binding of hepcidin to ferroportin and cause non-classical ferroportin disease have been described in a family from Thailand and in patients from China [13, 21, 22].

## Clinical features and natural history

### Clinical manifestations

The clinical manifestations of HFE p.Cys282Tyr homozygous hemochromatosis were initially appreciated by Trousseau and von Recklinghausen [29, 30]. Early clinical cohort studies described significant morbidity and mortality [31–33]. Importantly, subjects without cirrhosis were shown to have survival equivalent to control populations [31, 34]. The disorder was shown to be inherited in an autosomal recessive fashion by Simon and colleagues [33], and in tight linkage disequilibrium with the HLA complex on chromosome 6p [35], followed later by discovery of the *HFE* gene by Feder et al. [36].

Following discovery of the *HFE* gene [36], population studies demonstrated variable biochemical and clinical manifestations [11, 24, 37, 38]. Cross-sectional cohort studies suggested that hemochromatosis was not associated with increased mortality [39–42]. More recent population studies have shown that males, but not females, homozygous for p.Cys282Tyr have a significantly increased mean risk of death by age 75 years of 19.5% compared to 15.1% for controls [43]. Compound or simple heterozygosity for p.Cys282Tyr and/or p.His63Asp was found not to be associated with increased risk of premature death [24, 25, 43, 44]. P.Cys282Tyr homozygosity was associated with excess dementia, delirium, sarcopenia, frailty, and chronic pain after the age of 60 years in males [45, 46].

Homozygosity for p.Cys282Tyr is associated with morbidity in up to 40% of males and 13% of females [11, 24, 25, 38, 47, 48]. The variable biochemical and clinical penetrance is most likely due to multiple genetic and environmental modifiers [49]. Males are likely at higher risk than females due to the absence of the protective effects of menstruation and pregnancy [50]. Symptoms are non-specific and often equally prevalent in individuals either with or without hemochromatosis [24, 37]. The commonest symptom is fatigue, which is observed mainly in males with elevated serum ferritin levels [24].

The most frequent significant clinical manifestations are liver disease and arthritis [5, 31, 34, 51, 52]. Males, but not females, who are homozygous for p.Cys282Tyr have a greater than fourfold increased risk of developing liver disease compared to those without *HFE* variants [25]. Male p.Cys282Tyr homozygotes also have increased risks of arthritis, colorectal cancer, pneumonia, and diabetes mellitus [25, 53]. P.Cys282Tyr homozygous females have a 1.3- to twofold increased risk of colorectal cancer, breast cancer, and arthritis compared to those without *HFE* gene pathogenic variants [25, 53].

#### Liver Disease

Advanced liver fibrosis or cirrhosis [54] in HFE-related hemochromatosis is rare under the age of 45 years in the absence of other liver co-morbidities, occurring in 8–25% of all HFE p.Cys282Tyr homozygotes [38, 51, 52, 55, 56]. Risk factors include excessive alcohol consumption, diabetes mellitus, arthritis, serum ferritin levels greater than 1000 µg/L, liver iron concentration greater than 200 µmol/g, and total mobilizable iron stores by therapeutic phlebotomy of greater than 9.6 g [47, 56–60].

HFE p.Cys282Tyr homozygous males have a 12-fold increased lifetime risk of primary liver cancer compared to those without *HFE* variants [43]. Females who are HFE p.Cys282Tyr homozygous are not at increased risk of liver cancer [43]. The greatest risk of primary liver cancer occurs in those with cirrhosis [31, 34, 61], and these individuals should be recommended to undergo routine 6-monthly liver ultrasound for liver cancer surveillance [5, 51, 52, 62]. Regression of cirrhosis to Scheuer grade F2 or less results in a reduction in the risk of liver cancer, although cirrhosis persists in the majority after treatment [61].

#### Arthritis

Hemochromatosis arthritis affects at least 24% of individuals and is a major cause of morbidity [63, 64]. Classically, arthropathy affects the metacarpophalangeal joints followed thereafter by hip, ankle, radiocarpal, elbow, shoulder and knee joints, as well as the lumbar spine [63, 64]. It can be challenging to discriminate between hemochromatosis arthropathy and degenerative osteoarthritis (also known as type 1 polyarticular osteoarthritis) [64]. It is unclear why arthropathy affects only a subset of people with hemochromatosis. Arthritis may occur at any point during the natural history of the disease, even following successful phlebotomy therapy [52, 64]. Risk factors for arthritis include increasing age, advanced liver fibrosis, serum ferritin levels elevated greater than 1000 µg/L, and elevated serum transferrin saturation ≥ 50% for prolonged periods of time [65, 66].

Liver disease and arthritis tend to occur concomitantly. Hemochromatosis arthritis is more likely with higher iron load or more advanced liver disease [67, 68]. A recent study showed that arthritis was strongly associated with advanced liver fibrosis, and 84% of HFE p.Cys282Tyr homozygous subjects with advanced hepatic fibrosis had arthritis, while 34% p.Cys282Tyr homozygotes with arthritis had advanced hepatic fibrosis. Importantly, only 5% of subjects without arthritis had advanced hepatic fibrosis; thus, the absence of arthritis had a 95% negative predictive value for advanced liver fibrosis [57].

#### Other clinical manifestations

Other conditions which have been reported in HFE p.Cys282Tyr homozygous hemochromatosis include diabetes mellitus, hyperpigmentation, hypogonadotropic hypogonadism, and cardiomyopathy [25, 51, 52, 62]. These conditions are usually managed as per standard of care, and in addition to the treatment of iron overload. Cardiomyopathy is a rare complication which is potentially reversible by iron removal therapy [69].

## Secondary iron overload

Secondary iron overload is often due to a combination of increased iron absorption and recurrent blood transfusions in the setting of hemoglobinopathies, such as thalassemia and sickle cell disease. In addition, increased hepatic iron stores can be seen in chronic liver disease, such as alcoholic liver disease, chronic hepatitis C, and metabolic-associated fatty liver disease (MAFLD)[70, 71].

The role of alcohol in the development of increased liver iron has been clarified in recent years. Alcohol can result in increased intestinal iron absorption due, in part, to alcohol-induced down-regulation of hepcidin expression and altered intestinal iron transporters [72]. In alcoholic subjects, Perls’ method of staining liver sections often reveals stainable iron in Kupffer cells probably reflecting iron release from damaged hepatocytes. Hemochromatosis subjects who consume greater than 60 g of alcohol per day are nine times more likely to develop cirrhosis than those who drink less than this amount [73].

Mild increases in liver iron stores can be seen in patients with manifestations of the metabolic syndrome. Such patients also have elevated serum ferritin concentration with normal or moderately increased transferrin saturation. This condition is known as the dysmetabolic iron overload syndrome [74]. It is worth noting that many subjects with MAFLD have elevated serum ferritin concentration with normal iron stores. Quantitative assessment of tissue iron by MRI scan is useful in these circumstances. There is no conclusive evidence to support the role of phlebotomy in patients with mild increases in liver iron stores or MAFLD. However, it is worth noting that hepatic stellate activation was detected in liver biopsies of hemochromatosis patients with a hepatic iron concentration of 60 µmol/g dry weight [75], suggesting that this modest degree of iron loading may be important in the setting of other hepatic co-toxins.

## Iron toxicity and hepatic fibrogenesis

### Iron toxicity

Abnormal iron homeostasis in HFE-hemochromatosis can lead to a wide variety of different iron-induced pathologies, including arthritis, cardiomyopathy, diabetes, cirrhosis, and HCC. Excessive iron deposition in the liver leads to the generation of reactive oxygen species (ROS), oxidative damage to intracellular organelle membranes and DNA strand breaks, and subsequent organelle dysfunction and liver cell injury [62, 76, 77], that can lead to cell death via apoptosis, necrosis, or iron-dependent ferroptosis [78, 79]. Both free iron and labile iron, including non-transferrin-bound iron, catalyze the formation of ROS via the Fenton and Haber–Weiss reactions, which can impact hepatic mitochondrial, microsomal, and lysosomal function [80–82]. Therefore, homeostatic regulation of intracellular iron via binding to ferritin or hemosiderin under normal physiological conditions is critical. Under conditions of excessive iron overload as seen in HFE-hemochromatosis, normal mechanisms of antioxidant defense that include glutathione peroxidase, catalase, and superoxide dismutase [83] are overwhelmed.

### Hepatic fibrogenesis

Iron-induced liver cell injury results in the release of cell damage-associated cytokines, chemokines, and proinflammatory mediators, which leads to hepatic inflammation [84], the activation of hepatic stellate cells into pathological collagen-producing myofibroblasts, and ultimately hepatic fibrosis, cirrhosis, and liver cancer [77]. In recent years there has been conjecture over the role of iron per sé in liver pathology as iron toxicity studies have largely been conducted on in vitro cultured liver cells with relatively scant in vivo evidence either in animal models of iron overload or in human HFE-hemochromatosis liver tissue [85]. Indeed, a recent proposal suggests that in conditions where iron is proposed to be pathogenic, iron-induced liver damage may in fact be potentiated by coexistent inflammation, with subsequent necrosis and hepatic stellate cell activation driving fibrosis [85]. While iron and transferrin have been shown to induce hepatic stellate cell activation and collagen expression in vitro [86–88], in vivo models of iron overload demonstrate only minor fibrosis, although the numbers of alpha-smooth muscle actin (αSMA)-positive myofibroblasts were significantly increased [89, 90]. In humans with HFE-hemochromatosis, studies have shown a significant positive correlation between the numbers of α-SMA-positive hepatic stellate cells and increasing hepatic iron concentration, suggesting a causal link [75]. However, that study demonstrated that in early disease these cells are located in zone 3 of the hepatic acinus, distal to the zone 1 iron-laden hepatocytes, implying hepatic stellate cells are activated into collagen-producing myofibroblasts by soluble mediators released from iron-damaged hepatocytes rather than by iron per sé [75, 85]. Regardless of the mechanisms responsible for liver pathology, hepatic fibrosis is reversible with iron removal via phlebotomy therapy [47], with the risk of developing liver cancer significantly diminished if fibrosis staging is reduced to F2 or less [61]. Human studies have shown that as the severity of liver fibrosis progresses, the number of hepatic progenitor cells increase, mimicking the pathological processes associated with the “wound healing” and carcinogenic response of the liver to injury which is observed in chronic viral hepatitis and alcohol-related liver disease [91, 92].

While HFE-hemochromatosis has traditionally been viewed as a non-inflammatory condition, there is growing evidence that suggests the disease does indeed encompass an inflammatory component. Tissue ferritin is released by iron-damaged hepatocytes and Kupffer cells in iron overload conditions and has been shown to act as an iron-independent proinflammatory mediator of hepatic stellate activation as a portent to fibrogenesis [84], acting via a high affinity binding protein for H-subunit ferritin (FTH) [93]. Serum ferritin levels have been demonstrated to be a better predictor of fibrosis severity than hepatic iron concentration, independent of gender, steatosis, or alcohol consumption [59], adding weight to the observations linking ferritin to hepatic stellate cell activation. Iron loading of hepatocytes in patients with HFE-hemochromatosis has been shown to lead to impaired hepatocyte replication and senescence, which was correlated with serum ferritin levels, hepatic iron concentration and oxidative stress, which stimulates the ductular reaction and fibrosis [91]. In that study, portal inflammation was shown to occur in HFE-hemochromatosis and was independently associated with the ductular reaction and fibrosis [91], demonstrating the potential mechanism of iron overload-induced injury and fibrogenesis in this condition. In HFE-hemochromatosis, it is the accumulation of excess Kupffer cell iron, which follows progressive and sustained accumulation of excessive iron within hepatocytes, that appears to be a necessary precursor or trigger for the development of hepatic fibrogenesis [94]. This being said, many patients with HFE-hemochromatosis do not develop severe fibrosis even in the presence of significant iron overload, which suggests that in addition to iron and inflammation, there remains a role for environmental modifiers, such as fat, excessive alcohol consumption and diabetes mellitus, as well as genetic modifiers, of the fibrogenic processes associated with this disease [49, 60, 95, 96], including gene variants of *PNPLA3* and *PCSK7* which have been shown to associate with increased liver disease risk [97–99].

It is in the context of the current knowledge outlined above that these clinical practice guidelines for the management of hemochromatosis have been developed to assist clinicians in the day-to-day care of affected patients and their relatives.

## Question 1: is general population screening for hemochromatosis indicated? If not, and in the absence of a relevant family history, then who should be tested for hemochromatosis?

General population screening for hemochromatosis has not been recommended due to variable and incomplete penetrance and a lack of any proof of resulting survival advantage [51, 52, 100, 101]. However, the recent report of significantly increased mortality in adult male p.Cys282Tyr homozygotes compared with those without HFE variants in the UK Biobank study supports re-examination of the utility of screening in susceptible male populations [43]. Screening is indicated in first-degree relatives of probands and is discussed in more detail later [51, 52].

Patients with symptoms, signs, or biochemical abnormalities that could be consistent with hemochromatosis should undergo measurement of serum iron indices. Fatigue is common and may be the only presenting symptom. Hemochromatosis should be considered in type 2 diabetic patients, and patients with loss of libido, unexplained cardiac failure or cardiac arrhythmias and significant polyarthropathy—particularly if the 2nd and 3rd metacarpophalangeal joints are involved. Abnormal liver function tests should prompt consideration of hemochromatosis, even if there is another diagnosis because MAFLD and alcoholic liver injury are common in patients with underlying hemochromatosis. On occasions, biochemical evidence of iron overload can be seen unexpectedly—for example, when considering iron deficiency as a cause of fatigue in menstruating females. These patients should be managed in the same manner as those with symptoms of iron overload.

### Recommendation:

**Fig. 2:**
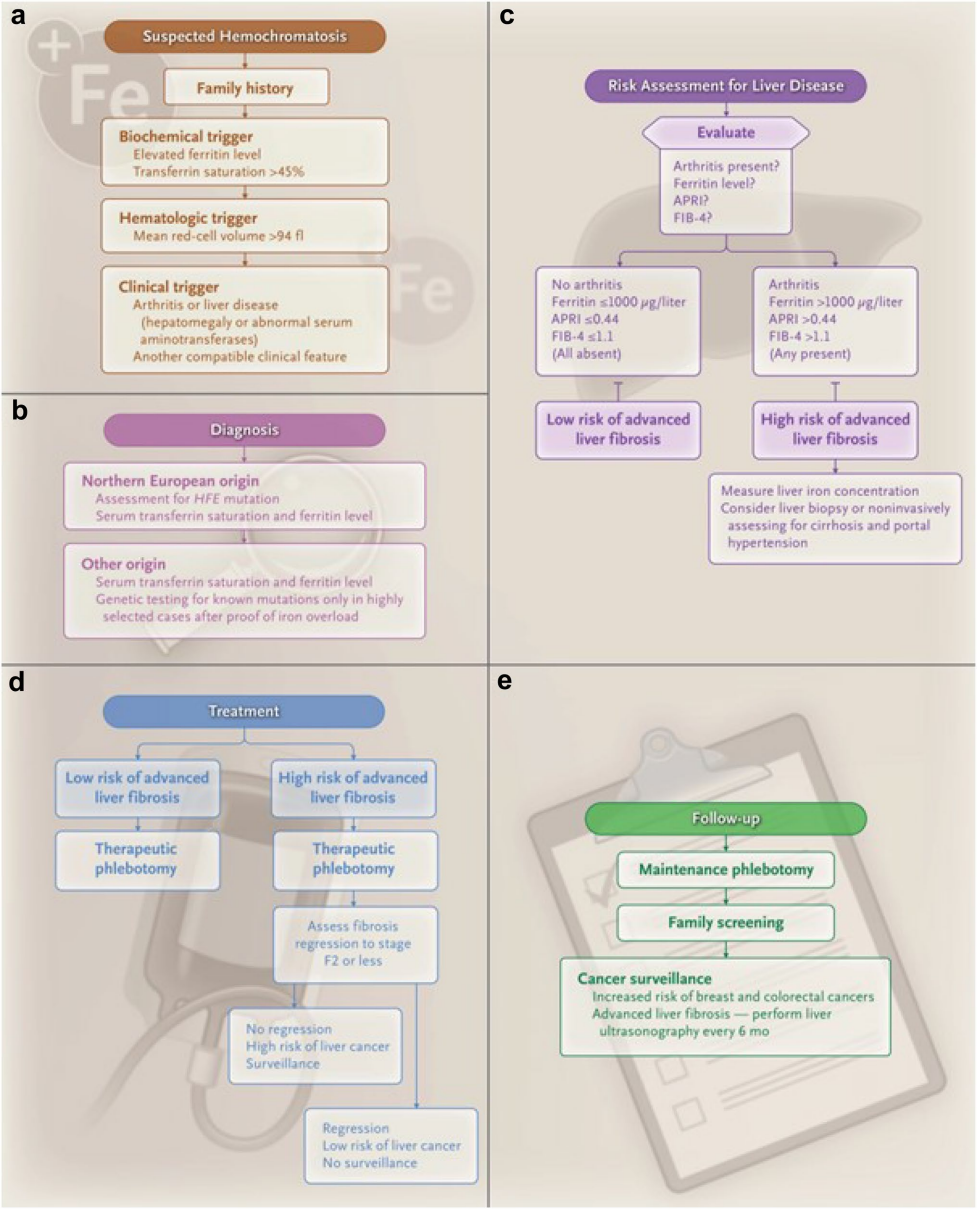
Recommended Key Steps in the Clinical Evaluation of Hemochromatosis. Features which raise the need to suspect hemochromatosis are shown in Panel A (it is possible that more than one of these may be present at any one time), how to diagnose hemochromatosis in Panel B (the arrows represent the choice of options to consider), and how to assess for liver disease in Panel C. Panel D shows treatment, and Panel E shows follow-up (the arrows represent the range of issues to consider). APRI denotes aspartate aminotransferase-to-platelet ratio index, and FIB-4 Fibrosis-4 index. From The New England Journal of Medicine, John K. Olynyk and Grant A. Ramm, Hemochromatosis, Volume 387:2159–70. Copyright © (2022) Massachusetts Medical Society. Reprinted with permission, https://www.nejm.org/doi/full/10.1056/NEJMra2119758 [95]

General population screening is not recommended but individuals of European descent with any clinical symptoms or signs compatible with the diagnosis or a family history of iron overload should be evaluated with genetic testing and measurement of serum transferrin saturation and ferritin levels. Patients with symptoms, signs, or biochemical abnormalities consistent with hemochromatosis should undergo measurement of serum ferritin concentration and transferrin saturation (HIGH QUALITY of EVIDENCE; STRONG RECOMMENDATION) (Fig. 2).

## Question 2: should screening be offered to first-degree relatives of patients with hemochromatosis? If so, what tests should be used and what age should screening for hemochromatosis take place? How frequently should hemochromatosis patients with normal serum ferritin at diagnosis be retested?

Because the carrier frequency of each of the two common HFE pathogenic variants, p.Cys282Tyr and p.His63Asp, is very high among people of European ancestry, screening should be offered to the first-degree relatives of individuals who have HFE-related hemochromatosis. In relation to children of affected individuals, options include offering testing to each child or testing the reproductive partner of the affected individual. If the reproductive partner has neither the p.Cys282Tyr nor the p.His63Asp variant, then their offspring do not need to have further testing as their risk of HFE-related hemochromatosis is low [102]. This latter approach is recommended where the couple have more than one child as it is likely that fewer family members will require testing.

We recommend that testing be offered from late adolescence onwards. Data from the ironXS high school screening program showed that individuals identified with HFE p.Cys282Tyr homozygosity in late adolescence are very unlikely to have dangerously high body iron levels as measured by serum ferritin [103]. By offering this testing in late adolescence, the at-risk individual can make an informed decision as to whether or not they wish to have this testing.

### Recommendation

We recommend that individuals found to be homozygous for the HFE p.Cys282Tyr pathogenic variant or compound heterozygous for p.Cys282Tyr/p.His63Asp with normal serum ferritin, should have measurements of serum ferritin and transferrin saturation repeated no more frequently than once a year and up to once every 5 years, especially if previously stable and not rising [51, 95]. While it is unlikely that serum ferritin would increase from the normal range to a dangerously high level in 12 months, clinical experience suggests that recommending testing beyond annually presents a higher risk of people forgetting to undertake this test.

Despite the observation of significant concordance of disease expression between siblings with hemochromatosis, we also recommend testing of first-degree relatives of subjects without phenotypic expression for HFE p.Cys282Tyr and p.His63Asp variants. Parents and siblings of an affected individual should be tested for the HFE p.Cys282Tyr and p.His63Asp variants (LOW QUALITY of EVIDENCE; STRONG RECOMMENDATION).

## Question 3: what biochemical iron parameters are best used to test patients for hemochromatosis?

While generalized population screening for iron overload is not recommended [104], patients with suspected iron overload are identified by elevated serum ferritin and transferrin saturation > 45% [52, 105, 106] followed by genetic testing for HFE p.Cys282Tyr and p.His63Asp. Screening using both serum ferritin and transferrin saturation will identify the majority of individuals who will go on to develop iron overload [107]. While widely used, both transferrin saturation and serum ferritin have limitations. Transferrin saturation has high biological variability and low sensitivity to detect HFE-related hemochromatosis [108] and a retrospective review of outpatient referrals for elevated serum ferritin found 64% of patients with a serum ferritin threshold over 1000 µg/L did not have iron overload on biopsy [109]. Age and gender should be considered when assessing elevation of serum ferritin. Serum ferritin increases steadily in males until the sixth decade of life, while serum ferritin is lower in females of all ages but increases sharply post menopause [110]. Other biochemical triggers that may lead one to suspect hemochromatosis include mean red cell volume > 94 fl, and altered liver enzymes and clinical triggers include hepatomegaly and arthritis (Fig. 2A). The sensitivity and specificity for MCV > 94 fL are 67% and 91% for females, and 50% and 89% for males.

### Recommendation

The best initial tests are serum ferritin and transferrin saturation followed by genetic testing if both are elevated. Liver biopsy, non-invasive measures of liver fibrosis (see Question7) or MRI can then be performed to assess level of hepatic iron loading (see Question 6) and liver fibrosis (HIGH QUALITY of EVIDENCE; STRONG RECOMMENDATION) (Fig. 2).

## Question 4: who should undergo genetic testing for the p.Cys282Tyr/p.His63Asp variants in HFE?

Genetic testing for HFE p.Cys282Tyr and p.His63Asp should be offered to all individuals of European descent found to have both raised serum ferritin and transferrin saturation, individuals with an isolated raised serum ferritin where no other cause is apparent [111], as well as first-degree relatives of individuals with HFE-related hemochromatosis (see Question 2). There are no clear values of serum ferritin and transferrin saturation that should trigger such genetic testing [106]. The upper end of the accepted reference range for serum ferritin levels in Australia is 620 µg/L in males. 220 µg/L and 370 µg/L are the upper end of reference range for premenopausal and postmenopausal females, respectively [112, 113]. A transferrin saturation over 45% in both males and females is considered elevated [114]. If biallelic HFE pathogenic variants are not identified, then testing of other genes associated with iron overload should be considered (see section on Genetics of Hemochromatosis and Question 5). A person with severe iron overload who is found to be compound heterozygous for HFE p.Cys282Tyr/p.His63Asp should be offered testing of other genes known to cause severe iron overload since this genotype generally results in either no iron overload or mild iron overload.

### Recommendation

Genetic testing for HFE p.Cys282Tyr and p.His63Asp should be offered to all individuals particularly those of European descent having both raised serum ferritin and transferrin saturation, individuals with an isolated elevated serum ferritin or transferrin saturation where there is no other cause identified, and first-degree relatives of individuals with HFE-related hemochromatosis (HIGH QUALITY of EVIDENCE; STRONG RECOMMENDATION) (Fig. 2).

## Question 5: who should be tested for non-*HFE* variants?

Patients with proven iron overload without *HFE* variants are occasionally encountered in clinical practice. It is essential to confirm the presence of hemochromatosis before pursuing non-*HFE* variants as abnormal serum iron indices are often due to other liver diseases, such as MAFLD [111]. It is also important to exclude other causes of secondary iron overload, including thalassemia or parenteral iron administration. It is advised that patients with confirmed — but unexplained — iron overload should be referred to a specialist in iron disorders for further assessment. Genetic testing for non-*HFE* variants is available in specialized centers (*HAMP, HJV, TFR2, TF, CP, BMP6, SCL40A1*) and discussions with personnel experienced in this field is important to evaluate the likelihood that any identified variants of genes of interest are pathogenic [13]. First-degree relatives of patients with non-*HFE* mutations should undergo testing.

### Recommendation

Patients with unexplained proven iron overload, particularly those of non-European descent, should be evaluated by a specialist in iron overload disorders, and results of genetic testing should be discussed with those experienced in the field to advise on pathogenicity of identified variants (MODERATE QUALITY of EVIDENCE; STRONG RECOMMENDATION).

## Question 6: how is liver iron concentration quantified in a suspected case of hemochromatosis?

Quantitation of liver iron concentration is important in the assessment of the risk of advanced liver fibrosis and primary liver cancer and should routinely be performed in all individuals with serum ferritin levels > 1000 µg/L, arthritis, or otherwise judged to be at risk due to clinical or biochemical features of liver disease.

Quantitation of liver iron concentration has historically been undertaken invasively via liver biopsy, although more recently reliable methods have been described for non-invasive measurement [5, 51, 52, 58, 115]. Practical non-invasive methods include retrospective calculation of the iron removed based on the number and volume of therapeutic phlebotomies undertaken to reduce the serum ferritin level to 50–100 µg/L, based on a 500 mL phlebotomy removing approximately 250 mg of iron [51, 94], as well as magnetic resonance imaging. A number of differing magnetic resonance methods of liver iron deposition and their correlations with liver biopsy biochemical measurements have been described [116–120]. Allowing for the heterogeneity of iron deposition [119, 121, 122], magnetic resonance imaging provides good clinical utility for quantification of iron overload [52, 116, 119]. Magnetic resonance imaging is also accurate for quantification of myocardial iron deposition [123]. There are numerous different MRI methods for quantification of liver iron concentration [124, 125] some of which can be confounded by the presence of steatosis, fibrosis, or inflammation. The FDA-approved spin-density projection-assisted R2-MRI method of liver iron measurement (FerriScan®) has been shown to give results unconfounded by the presence of steatosis, stage of fibrosis, and grade of necroinflammation [119, 126, 127]. Of the published magnetic resonance methods for measuring liver iron concentration, only that described by St Pierre et al. [119] has been approved for human use by regulatory authorities in the United States of America, Europe, and Australia.

### Recommendation

All subjects with hemochromatosis and serum ferritin levels elevated to > 1000 µg/L should undergo measurement of hepatic iron concentration using available validated methods, including MRI, as described above and chemical estimation [128] (HIGH QUALITY of EVIDENCE; STRONG RECOMMENDATION).

## Question 7: how is hepatic fibrosis assessed in hemochromatosis?

The staging of hepatic fibrosis at diagnosis is necessary to assess for severity of liver injury and end organ damage in order to better guide clinical management, and in those with advanced fibrosis or cirrhosis, to screen for primary liver cancer. Assessment of progressive fibrosis is clearly warranted, as is regression of advanced fibrosis with phlebotomy therapy [47], which can guide the requirement for ongoing HCC surveillance. Recent evidence shows regression of biopsy-proven advanced fibrosis and cirrhosis with iron removal [61, 129], where 70% of patients with F3 and 20% of patients with F4 fibrosis showed regression of fibrosis stage over a median follow-up of 9.5 years [61]. The amount of iron removed via phlebotomy, or ‘mobilizable iron,’ to achieve iron depletion (reflected in a resultant serum ferritin < 100 µg/L) has been shown to correlate with the severity of fibrosis, with a recent study demonstrating that advanced fibrosis can be predicted above a threshold of 9.6 g of iron removed, with an area under the receiver operating characteristic (AUROC) curve of 0.92 or an hepatic iron concentration above 200 µmol/g (AUROC 0.83) [47, 56, 130]. Thus, understanding the potential burden of mobilizable body iron stores via phlebotomy may permit better stratification of patients requiring more sophisticated and costly assessment of liver disease complications [56].

In HFE-hemochromatosis, liver disease and arthritis can occur concurrently [95], with arthritis more likely in patients with higher body iron load and/or advanced hepatic fibrosis [67, 68]. Arthritis was recently shown to be strongly associated with the presence of advanced fibrosis, with 84% of patients with advanced fibrosis also having arthritis [57]. Of interest, the absence of arthritis had a 95% negative predictive value for advanced fibrosis [57].

Liver biopsy remains the ‘gold standard’ to assess for hepatic pathology in HFE-related hemochromatosis [51], although it is now used infrequently. If cirrhosis is suggested via clinical examination or ultrasound, a liver biopsy may not be recommended [106]. Elevated serum ferritin levels > 1000 µg/L can assist in identifying 20–45% of patients who would benefit from a liver biopsy [58, 131, 132], with several studies showing < 2% of HFE-hemochromatosis patients with ferritin < 1000 µg/L at diagnosis have bridging fibrosis or cirrhosis in the absence of other co-morbidities, with a 94% negative predictive value [58, 131]. The addition of elevated aspartate aminotransferase and a platelet count < 200 × 10^9^/L to a serum ferritin > 1000 µg/L has been reported to predict cirrhosis in 80% of patients [58]. However, in another study, application of this algorithm did not detect 30% of cirrhotic patients [133]. Rather, that study demonstrated that elevated serum hyaluronic acid > 46.5 ng/mL predicted 100% of patients with cirrhosis and when using a serum ferritin > 1000 µg/L to triage those for biopsy, obviating the need for a liver biopsy in 60% of patients with HFE-hemochromatosis [133]. Measurement of hyaluronic acid is not used in routine clinical practice limiting its potential utility, and others have reported its relative lack of diagnostic accuracy, albeit in a study limited only to patients with a serum ferritin > 1000 µg/L [134].

Far fewer liver biopsies are performed in the modern era with the advent of non-invasive modalities to quantify hepatic iron [119, 135, 136], and to detect and stage liver disease, thus prospective studies to assess the utility of contemporary non-invasive technologies matched against liver biopsy-staged fibrosis in HFE-hemochromatosis are increasingly unlikely. Many newer non-invasive methods to detect and stage liver disease, such as direct and indirect serum fibrosis biomarker panels and elastography, have been validated in chronic viral hepatitis or MAFLD (reviewed in [137]), and a few such studies are also emerging in cohorts of patients with HFE-hemochromatosis.

A study by Legros and colleagues demonstrated the potential utility of transient elastography to detect advanced fibrosis in patients with HFE-hemochromatosis [134]. In patients with a serum ferritin > 1000 μg/L, a liver stiffness measurement < 6.4 kPa was proposed to exclude advanced fibrosis, whereas a value > 13.9 kPa was predictive of advanced fibrosis [134]. While transient elastography is recommended in recent clinical guidelines [106], caution should be exercised as that study only included patients with raised transaminases or a serum ferritin > 1000 μg/L, with only 15 patients having advanced fibrosis and the proposed elastography cut-offs only correctly diagnosing 61% of patients in the study. Of interest using these cut-off values restricted the requirement of liver biopsy to 39% of patients with indeterminate or invalid liver stiffness measurements, which was a similar observation to a previous study using a combination of elevated serum ferritin and serum hyaluronic acid [133].

Hepascore (which uses an algorithm based on age and gender, and serum levels of γ-glutamyl transpeptidase (GGT), hyaluronic acid, bilirubin, and α2-macroglobulin [138, 139]) and transient elastography were assessed in a study of HFE-hemochromatosis patients without matched liver biopsy-staged fibrosis [140]. This study used cut-offs for advanced fibrosis based on biochemical panels (as discussed below) and showed that advanced fibrosis could be detected but only in those with a serum ferritin > 1000 µg/L. Once again caution in interpretation is required as this study was not matched to liver biopsy-validated fibrosis staging.

As mentioned, a recent study has evaluated the utility of simple, inexpensive biochemical panels that can be determined via routine liver function tests in patients with HFE-hemochromatosis [141]. In that study, serum from 181 patients with HFE-hemochromatosis who had undergone liver biopsy for their clinical management were assessed for aspartate aminotransferase-to-platelet ratio index (APRI), fibrosis-4 (FIB-4), and gamma-glutamyl transferase (GGT)-platelet ratio (GPR). An APRI score > 0.44 and a FIB-4 score > 1.1 were both demonstrated to detect liver biopsy-validated advanced fibrosis with 81% diagnostic accuracy [141]. These data indicate that thresholds for the diagnosis of advanced fibrosis in HFE-hemochromatosis are lower than those observed for more overtly inflammatory chronic liver diseases, such as chronic viral hepatitis, alcoholic liver disease, and MAFLD [106]. That study recommended that patients who do not meet the threshold for advanced fibrosis should proceed to therapeutic phlebotomy, while in those patients who breached these thresholds, a liver biopsy should be performed to confirm advance fibrosis, and if present, these patients should undergo routine surveillance for complications, such as oesophageal varices and HCC [141]. The study also followed 64 patients after de-ironing via phlebotomy and demonstrated a significant decrease in APRI, FIB-4, and GPR in patients across the spectrum of fibrosis staging, and in a subset with available post-treatment liver biopsies, decreased APRI and GPR scores reflected fibrosis regression [141]. Finally, that study showed that assessing APRI post-phlebotomy predicted that 87% of patients with advanced fibrosis at diagnosis decreased to APRI levels indicative of mild F1–F2 fibrosis [141], suggesting that routine assessment of APRI may be clinically useful for monitoring the regression of fibrosis with treatment. These data require validation by others but such non-invasive biomarker panels show promise at least as a screening tool to determine the requirement for subsequent liver biopsy assessment of advanced fibrosis [95].

### Recommendation

Liver biopsy should be considered in patients with verified HFE-hemochromatosis to assess for advanced hepatic fibrosis if any of the following non-invasive markers are exceeded:

Serum ferritin > 1000 µg/L (HIGH QUALITY of EVIDENCE; STRONG RECOMMENDATION); hepatic iron concentration > 200 µmol/g by MR methods (MODERATE QUALITY of EVIDENCE; STRONG RECOMMENDATION); APRI > 0.44; FIB-4 > 1.1; mobilizable iron stores > 9.6 g (MODERATE QUALITY of EVIDENCE; STRONG RECOMMENDATION); and transient elastography liver stiffness measurement > 13.9 kPa (HIGH QUALITY OF EVIDENCE; STRONG RECOMMENDATION) (Fig. 2).

Transient elastography < 6.4 kPa predictive of the absence of advanced hepatic fibrosis (LOW QUALITY of EVIDENCE; WEAK RECOMMENDATION), a serum ferritin < 1000 µg/L (HIGH QUALITY of EVIDENCE; STRONG RECOMMENDATION), and the absence of arthritis (MODERATE QUALITY of EVIDENCE; STRONG RECOMMENDATION) are non-invasive assessments most predictive for the absence of advanced hepatic fibrosis in HFE-hemochromatosis.

## Question 8. how should patients with HFE p.Cys282Tyr/p.His63Asp compound heterozygosity or p.His63Asp homozygosity be managed?

It is very uncommon for patients with p.Cys282Tyr/p.His63Asp compound heterozygosity to develop severe iron loading and studies have also shown that iron overload is rare in individuals homozygous for p.His63Asp [142]. As such, it is important to accurately evaluate the phenotype of such subjects and identify factors that may alter serum iron indices and/or increase hepatic iron stores. One can consider testing of genes other than HFE in patients with these variants and severe iron overload—although that should occur in specialized centers.

The management of these individuals is determined by their phenotype and phlebotomy commenced if iron overload is proven. Risk factors for liver disease and secondary iron overload should be addressed, including attention to alcohol consumption and obesity [73, 143]. Patients who are identified as p.Cys282Tyr/p.His63Asp compound heterozygotes but without iron overload can undergo routine monitoring of their iron indices although in practice their chance of developing significant iron overload is low. It is unclear if these subjects with mild iron overload benefit from phlebotomy—but it is likely that they will have the same gain in quality of life as p.Cys282Tyr homozygotes with mild iron overload [144].

### Recommendation

It is important to accurately evaluate the phenotype of p.Cys282Tyr/p.His63Asp compound heterozygous subjects and identify factors that may alter serum iron indices and/or increase hepatic iron stores or cofactors for liver disease expressivity.

Annual monitoring with measurements of serum ferritin and transferrin saturation of subjects with p.Cys282Tyr/p.His63Asp compound heterozygosity or p.His63Asp homozygosity and normal iron indices is acceptable recognizing the probability of developing significant iron overload is low. Subjects with p.Cys282Tyr/p.His63Asp compound heterozygosity or p.His63Asp homozygosity and mild iron overload are unlikely to develop significant iron overload but can be managed with phlebotomy if symptomatic. Annual monitoring of iron studies in these subjects is recommended if phlebotomy is not commenced. Patients with p.Cys282Tyr/p.His63Asp compound heterozygosity or p.His63Asp homozygosity with significant iron loading should be managed with phlebotomy and referred to a specialized center for possible testing for mutations in related genes (HIGH QUALITY of EVIDENCE; STRONG RECOMMENDATION).

## Question 9: at what level of serum ferritin should therapy with phlebotomy commence? What is the goal of phlebotomy treatment and how frequently should biochemical indices be monitored?

Whilst most laboratories report that serum ferritin concentration is elevated if the value is > 300 µg/L in males and 200 µg/L in females, it is well recognised that serum ferritin concentration varies with age as well as gender [110]. Therefore, consideration of these factors, the transferrin saturation, and the variable phenotype are key to the interpretation of iron studies, and by inference, the need for phlebotomy in patients with hemochromatosis.

Despite some minor variations, there is general consensus between international guidelines on the core aspects of management of patients with hemochromatosis [52, 106]. All guidelines agree that excess iron should be removed by phlebotomy, and treatment should be commenced early with an initial de-ironing phase followed by a maintenance phase to keep serum ferritin concentration in the low-normal range. Phlebotomy does not need to commence in adult p.Cys282Tyr/p.His63Asp homozygous patients, with a serum ferritin < 300 µg/L in male patients and 200 µg/L in female patients—as many of these patients will not subsequently develop significant iron overload. Monitoring of iron studies and liver function tests on an annual basis is appropriate.

Phlebotomy is recommended in patients with elevated serum ferritin concentration. Studies have shown that patients who have a serum ferritin concentration of > 1000 µg/L are at a high risk of hepatic cirrhosis and require investigation to determine the extent of hepatic fibrosis, as well as commence venesection therapy [108, 131, 133]. Patients with serum ferritin concentration above the reference range but less than 1000 µg/L are at risk of progressive iron accumulation and subsequent target organ damage and should undergo phlebotomy. A blinded study comparing reduction of body iron stores versus sham treatment demonstrated benefit for those with such iron indices [144].

The goal of phlebotomy is to reach a target serum ferritin of 50 µg/L in the induction (de-ironing) phase and maintain a serum ferritin concentration of 50–100 µg/L during the maintenance phase. In the initial de-ironing phase, 500 mL of blood can be removed on a weekly basis if tolerated by the patient. On occasions the amount can be increased to 1000 mL or reduced to 250 mL depending on the patient’s tolerance, and likewise the frequency can be reduced to fortnightly. The frequency of phlebotomy in the maintenance phase varies but is generally required every 2–4 months. The target serum ferritin concentration can be relaxed somewhat if the patient is finding difficulties tolerating such a low serum ferritin concentration. Despite being an effective and safe therapy, some patients find difficulty complying with venesection due to a variety of causes, including lack of motivation, needle phobia, difficult venous access, and concomitant iron loading anemias [145]. Careful alterations to venesection protocols or addition of chelation therapy can be considered.

It is important to monitor hemoglobin concentration and serum ferritin during both the induction phase and maintenance phase. Measurement of hemoglobin at each phlebotomy is recommended, whereas serum ferritin can be measured at every fourth phlebotomy during the early induction phase but increased to every phlebotomy when the serum ferritin reaches 200 µg/L. Serum ferritin should be monitored 2–3 times per year during the maintenance phase and the frequency of phlebotomy adjusted accordingly. Non-compliance with venesection is more common in the maintenance phase and such patients are at risk of reaccumulating iron and associated complications [145].

Unexplained reductions in the need for phlebotomy should be investigated as occult blood loss may be an underlying cause. On occasions, no explanation for the reduced need can be identified, suggesting that there may be some intra-individual variation in phenotypic expression [146].

### Recommendation

Patients with elevated serum ferritin concentration should commence a venesection program. The generally accepted target range for ferritin is 50 µg/L in the de-ironing phase and 50–100 µg/L in the maintenance phase. During the induction phase, measurement of hemoglobin at every venesection and serum ferritin at every fourth venesection is recommended until the serum ferritin concentration reaches 200 µg/L after which serum ferritin should be measured at each venesection. During the maintenance phase, measurements of serum ferritin are recommended 2–3 times per year and venesection schedule adjusted accordingly (HIGH QUALITY of EVIDENCE; STRONG RECOMMENDATION) (Fig. 2).

## Question 10: when should erythrocytapheresis be considered?

Erythrocytapheresis is a method of red blood cell removal where whole blood is drawn from the patient, centrifuged to separate whole blood into plasma and red cells and then plasma is returned to the individual. Therefore, important blood components, including plasma proteins, clotting factors, and platelets, are returned to the individual being treated. Studies have shown that erythrocytapheresis can normalize serum ferritin in a shorter timeframe with fewer procedures than phlebotomies and is less likely to result in symptoms of hypovolemia [147–149]. This procedure can remove up to four times as many red cells per treatment than phlebotomy [150]. Disadvantages include that erythrocytapheresis requires specialized equipment and expert staffing that is not universally available and, that where it is available, it may be considerably more distant from a person’s residence than a venue where phlebotomy can be performed. In addition, erythrocytapheresis is more expensive than phlebotomy.

### Recommendation

Erythrocytapheresis should be considered for individuals who have problems with symptoms from hypovolemia from phlebotomy, those with cardiac morbidity, hypoproteinemia, and/or thrombocytopenia [150] (MODERATE QUALITY of EVIDENCE; STRONG RECOMMENDATION).

## Question 11: what is the role of iron chelation in hemochromatosis?

Iron chelation is an alternative to phlebotomy therapy for those who cannot tolerate the procedure for medical or personal reasons. Chelation therapy may take the form of oral or parenteral approaches. Of these approaches, oral deferasirox is probably the best tolerated of the options [151]. Alternatively, erythrocytapheresis may be undertaken (see Question 10 above).

### Recommendation

Iron chelation is an alternative to phlebotomy/erythrocytapheresis therapy for those who cannot tolerate the procedure for medical or personal reasons (LOW QUALITY of EVIDENCE; STRONG RECOMMENDATION).

## Question 12: how is juvenile hemochromatosis best managed?

Juvenile hemochromatosis is an autosomal recessive disorder which manifests usually under the age of 30 years with significant iron overload. It is rare and falls under the classification of non-HFE-hemochromatosis. It can be caused by homozygous mutations in *HJV* or *HAMP,* which both result in loss of production of hepcidin and subsequent iron overload [152, 153]. It is best managed by early diagnosis and phlebotomy therapy to reduce body iron stores [5].

### Recommendation

Individuals presenting with iron overload less than 30 years of age should undergo genetic testing for *HFE-* and non-*HFE*-hemochromatosis. Accurate assessment of the degree of iron overload affecting the liver and heart followed by early phlebotomy treatment offers the best prognosis (MODERATE QUALITY of EVIDENCE; STRONG RECOMMENDATION).

## Question 13: what dietary modifications are recommended in hemochromatosis?

Many patients with hemochromatosis have hepatic co-morbidities, including MAFLD and alcoholic liver disease—both of which can accelerate progression of the underlying liver disease [73, 143]. In this context, it is recommended that affected patients adopt healthy lifestyles following appropriate recommendations around dietary intake and alcohol ingestion aiming to maintain a normal body mass index [114]. Dietary iron intake, for example, by ingesting red meat, does not need to be restricted, but iron-containing supplements should be avoided. Black tea and non-citrus fruit may possibly reduce iron accumulation [154, 155]. It has been suggested that vitamin C can increase iron absorption and worsen the hemochromatosis phenotype. Limiting the intake of vitamin C supplementation to the recommended daily intake would seem appropriate [51, 156]. *Vibrio vulnificus* is a pathogen that can contaminate seafood (e.g., oysters) and hemochromatosis patients with high levels of circulating iron may be susceptible to life-threatening infections following exposure [157, 158].

### Recommendation

Patients with hemochromatosis should adopt a healthy lifestyle, including maintaining normal body weight and limiting alcohol consumption [114]. Dietary iron intake does not need to be restricted, but iron supplements should be avoided (MODERATE QUALITY of EVIDENCE; STRONG RECOMMENDATION).

## Question 14: when should patients with hemochromatosis undergo surveillance for HCC?

Patients with cirrhosis due to underling hemochromatosis have a 100-fold increased risk of developing HCC. Surveillance using six-monthly ultrasounds has been shown to lead to earlier diagnosis and improved survival in patients with cirrhosis from other causes of liver disease [159–162]. It is likely that these benefits extend to patients with hemochromatosis and cirrhosis. In general, surveillance should only be offered to patients who would consider treatment options for a newly diagnosed HCC and not offered to those with limited life expectancy. The benefits of measuring alpha-fetoprotein concentration in addition to ultrasound remains unclear across a range of liver diseases, including hemochromatosis. Regression of cirrhosis to Scheuer grade F2 or less with phlebotomy therapy is associated with a reduction in the risk of liver cancer, although the majority of cases of cirrhosis persist after treatment [61]. Where such regression is proven, clinicians may consider cessation of surveillance for HCC.

### Recommendation

In patients with cirrhosis due to hemochromatosis, six-monthly surveillance with ultrasound, with or without alpha-fetoprotein testing is recommended. Surveillance should only be undertaken if a diagnosis of HCC will alter management (HIGH QUALITY of EVIDENCE; STRONG RECOMMENDATION) (Fig. 2).

## Question 15: what is the therapeutic role of hepcidin or hepcidin mimetics?

Venesection therapy for hemochromatosis remains the treatment of choice, with iron depletion preventing organ dysfunction if commenced early. However, compliance and tolerance issues limit its suitability in some patients. Recent evidence on hepcidin modulation in animal models and development of hepcidin mimetics/agonists has demonstrated their potential use in therapy for hemochromatosis and other iron overload disorders.

A recent clinical trial examining the hepcidin mimetic, Rusfertide (PTG-300), for use in hereditary hemochromatosis demonstrated reduction in both transferrin saturation and serum iron during treatment, reduced requirement for phlebotomy during the study period, and resulted in control of hepatic iron concentration [163]. Additional phase 2 trials have been undertaken in both transfusion-dependent and non-transfusion-dependent thalassemia patients [164]. PTG-300 was efficacious in reducing serum iron and transferrin saturation with mild to moderate adverse events. A synthetic endogenous human hepcidin (LJPC-401) also significantly reduced serum iron but the study did not examine tissue iron concentrations. The use of mini-hepcidins in preclinical animal models of non-transfusion-dependent thalassemia are more promising, but there are currently no active clinical trials for mini-hepcidins [165].

Of interest, Chen et al. [166] demonstrated that hepcidin overexpression in animal models of hepatic steatosis results in attenuation of steatosis, indicating that patients with both iron overload and hepatic steatosis may benefit from hepcidin-based therapy.

### Recommendation

There are insufficient data to recommend the use of hepcidin-based therapy until further clinical trial data are available (HIGH QUALITY of EVIDENCE; STRONG RECOMMENDATION).

## Question 16: are patients with hemochromatosis suitable for liver transplantation and what is the risk of hemochromatosis recurrence after liver transplantation?

Hemochromatosis is a very uncommon indication for liver transplantation comprising only about 1% of all transplants, despite a prevalence of 1:200–400 in Caucasian populations. The discordance between the low number of patients undergoing liver transplantation and the high population prevalence is due to the variable phenotype of the condition with only a small percentage of patients developing sufficiently high liver iron stores to cause cirrhosis, as well as better clinician and community awareness resulting in early diagnosis and therapy. Many patients who proceed to liver transplantation have associated hepatic co-toxicities, such as alcoholic liver disease, MAFLD, or chronic viral hepatitis [167]. Initially, it was thought that patients with hemochromatosis had more adverse outcomes than other groups. This was largely due to the high number of patients who underwent liver transplantation for complicating hepatocellular carcinoma prior to the development of the Milan criteria developed by Mazzaferro et al., [168], as well as higher rates of infectious and cardiovascular complications [168]. Recent studies have shown similar survival and outcomes compared to other causes of liver disease [167, 169]. There is clear evidence that liver transplantation alleviates the underlying pathophysiological defect in hemochromatosis by restoring hepcidin levels to normal and re-establishing normal iron metabolism [169]. Thus, re-accumulation of iron post-liver transplant is most unusual and alternative causes should be considered if this occurs.

### Recommendation

Liver transplantation is an appropriate therapy for patients with decompensated liver disease and /or HCC. The risk of recurrence of hepatic iron loading is very low after liver transplantation and alternative causes should be considered should it occur (HIGH QUALITY of EVIDENCE; STRONG RECOMMENDATION).

## Question 17: how should pregnant hemochromatosis patients be managed?

It is important to avoid iron deficiency in subjects with known hemochromatosis undergoing venesection who wish to fall pregnant given its adverse effects on pregnancy outcomes [106]. Thus, iron studies should be closely monitored in this situation and venesection adjusted accordingly. Iron studies should be monitored during pregnancy and clinicians should be aware that pregnancy removes about 1 g of iron from the mother [114]. Thus, phlebotomy is usually ceased during pregnancy without adverse outcomes as the likelihood of rapid iron re-accumulation and associated iron toxicity is very low.

### Recommendation

Iron studies should be monitored in hemochromatosis patients wishing to fall pregnant and iron deficiency avoided. In general, phlebotomy can be ceased during pregnancy with iron studies continuing to be monitored (LOW QUALITY of EVIDENCE; STRONG RECOMMENDATION).

## Data Availability

Data is available on request.
